# Implementation of Delirium Screening at Scale in Older Patients With Emergency Hospital Admission

**DOI:** 10.1001/jamainternmed.2025.1128

**Published:** 2025-05-27

**Authors:** Emily Louise Boucher, Jasmine Ming Gan, Nicola G. Lovett, Sarah C. Smith, Sasha Shepperd, Sarah Tamsin Pendlebury

**Affiliations:** 1 Wolfson Centre for Prevention of Stroke and Dementia, Wolfson Building, Nuffield Department of Clinical Neurosciences, University of Oxford, United Kingdom; 2 Department of Acute General (Internal) Medicine, Oxford University Hospitals NHS Foundation Trust, United Kingdom; 3 Department of Geratology, Oxford University Hospitals NHS Foundation Trust, United Kingdom; 4 Nuffield Department of Population Health, University of Oxford, United Kingdom; 5 NIHR Biomedical Research Centre, Oxford University Hospitals NHS Foundation Trust, United Kingdom

## Abstract

This cohort study assesses the frequency of screen completion and the prevalence and outcomes of delirium diagnosis for older patients with unplanned admission to acute general (internal) medicine.

International guidelines recommend routine delirium screening for older hospital patients to improve care and outcomes.^1^ However, recognition and documentation of delirium is poor in routine practice.^2^ Large-scale implementation of delirium screening using validated tools is therefore warranted.

We previously developed a pragmatic cognitive screen including the Confusion Assessment Method (CAM)^3^ for delirium and the 10-point Abbreviated Mental Test (AMT)^4^ for use by resident physicians. Subsequently, physician-delivered screening for delirium as part of standard care was implemented by integrating the cognitive screen as a mandatory task in the electronic health record (EHR). In this cohort study, we assessed the frequency of screen completion and the prevalence and outcomes of delirium diagnosis in older patients with emergency hospital admission.

## Methods

The electronic cognitive screening form was triggered automatically on admission in all hospitals providing acute secondary care in Oxfordshire, UK, for all patients 70 years or older with emergency hospitalization and was completed by the resident physician (eMethods, eFigure 1 in Supplement 1). The screen included the AMT (range, 0-10 points; with scores ≤8 indicating impairment) to identify the presence and severity of cognitive impairment from any cause; and a question with the answer of yes, no, or uncertain regarding delirium diagnosis: “Does the patient have delirium?” based on the CAM.

The answer uncertain (indicating possible delirium) was designed to reflect that clinicians may be unsure if all CAM criteria are met (eMethods in Supplement 1). Cognitive, clinical, and demographic data were extracted from the EHR by the hospital information analysts and transferred by the research team into the Oxford Cognitive Comorbidity, Frailty and Ageing Research Database-Electronic Patient Records (ORCHARD-EPR)^5^ for analysis (eTable, eFigure 2 in Supplement 1). Data were restricted to first admissions to acute general (internal) medicine between January 1, 2017, and December 31, 2019, covering the period from full EHR implementation up to the COVID-19 pandemic. Data were censored on December 31, 2020. We calculated the prevalence of certain and uncertain delirium and odds ratios (ORs) for binary outcomes adjusted for age, sex, Charlson Comorbidity Index, and illness severity (eMethods in Supplement 1). ORCHARD-EPR is approved by the South Central Oxford C Research Ethics Committee for any relevant research led by the corresponding author without the need for further approval. We followed the STROBE reporting guideline. Data were analyzed using R version 4.2.1 (R Foundation for Statistical Computing). Statistical significance was set at 2-sided *P* < .05.

## Results

In 18 870 admissions (mean [SD] age, 82.9 [7.4] years; female, 52%), 77% had a cognitive screen completed. Certain delirium was present in 14% (95% CI, 13%-15%) and uncertain delirium in 20% (95% CI, 19%-20%) of admissions (Table 1). Compared with no delirium (783 of 9570 [8.2%]), in-hospital mortality was similarly increased for certain delirium (329 of 2020 [16%]; adjusted OR [AOR], 1.81; 95% CI, 1.56-2.09; *P* < .001) and uncertain delirium (427 of 2833 [15%]; AOR, 1.63; 95% CI, 1.43-1.86; *P* < .001) (Table 2). Length of stay greater than 7 days was also higher for certain delirium (945 of 2020 [47%]; AOR, 1.71; 95% CI, 1.55-1.88; *P* < .001) and for uncertain delirium (1201 of 2833 [42%]; AOR, 1.43; 95% CI, 1.31-1.56; *P* < .001) vs no delirium (3068 of 9570 [32%]). Delayed discharge and discharge destination other than usual residence were more likely in certain and uncertain delirium vs no delirium with effect sizes slightly larger for certain delirium. Readmission within 30 days of discharge was not increased for either group.

**Table 1.** Characteristics of Acute General Medicine Patients With a Completed Cognitive Screen by Delirium Status

Characteristic	Patients, No./total No. (%)				*P* value^a^	*P* value^b^
	ORCHARD-EPR (n = 14 572)	Delirium				
		Certain (n = 2047)	Uncertain (n = 2866)	No (n = 9659)		
Age, mean (SD), y	83.4 (7.2)	85.0 (7.0)	84.7 (7.1)	82.6 (7.2)	<.001	.12
Sex						
Female	7623/14 572 (52)	1141/2047 (56)	1600/2866 (56)	4882/9659 (51)	<.001	.95
Male	6949/14 572 (48)	906/2047 (44)	1286/2866 (44)	4777/9659 (49)		
CCI, mean (SD)	11.4 (10.7)	14.4 (11.7)	14.4 (11.5)	9.8 (9.9)	<.001	.73
Frailty markers						
Modified HFRS, mean (SD)	8.2 (6.1)	11.0 (6.5)	10.5 (6.3)	6.9 (5.5)	<.001	.01
Fall history	5360/12 053 (44)	899/1701 (53)	1244/2292 (54)	3217/8060 (40)	<.001	.39
Incontinence	3088/12 101 (26)	688/1729 (40)	908/2323 (39)	1492/8049 (19)	<.001	.67
Braden score, mean (SD)^c^	17.5 (3.6)	16.0 (3.5)	16.1 (3.7)	18.3 (3.3)	<.001	.14
Observations and laboratory results						
NEWS ≥5	3298/14 502 (23)	530/2031 (26)	714/2842 (25)	2054/9629 (21)	<.001	.44
CRP >6 mm/L	10740/13 635 (79)	1690/1976 (86)	2199/2721 (81)	6851/8938 (77)	<.001	<.001
Serum sodium <135 mm/L	4586/14 454 (32)	688/2035 (34)	789/2836 (28)	3109/9583 (32)	<.001	<.001
Infection diagnosis	1214/14 572 (8.3)	238/2047 (12)	253/2866 (8.8)	723/9659 (7.5)	<.001	.001

Abbreviations: CCI, Charlson Comorbidity Index; CRP, C-reactive protein; HFRS, Hospital Frailty Risk Score; NEWS, National Early Warning Score; ORCHARD-EPR, Oxford Cognitive Comorbidity, Frailty, and Ageing Research Database-Electronic Patient Records.

SI conversion factors: To convert CRP to mg/L, multiply by 10; serum sodium to mEq/L, multiply by 1.0.

^a^ Pearson χ^2^ test or Kruskal-Wallis test for no, uncertain, and certain delirium.

^b^ Pearson χ^2^ test or *t* test for uncertain vs certain delirium.

^c^ Range, 6-23, with high risk as a score ≥12.

**Table 2.** Outcomes of Certain and Uncertain Delirium vs No Delirium

Outcome	No delirium		Certain delirium			Uncertain delirium		
	Events, No./total No. (%)	Unadjusted OR (95% CI)	Events, No./total No. (%)	Unadjusted OR (95% CI)^a^	AOR (95% CI)	Events, No./total No. (%)	Unadjusted OR (95% CI)^a^	AOR (95% CI)
Death								
In hospital	783/9570 (8.2)	1 [Reference]	329/2020 (16)	2.18 (1.90-2.51)	1.81 (1.56-2.09)	427/2833 (15)	1.99 (1.75-2.26)	1.63 (1.43-1.86)
30 d	490/8787 (5.6)	1 [Reference]	147/1691 (8.7)	1.61 (1.33-1.95)	1.35 (1.11-1.64)	237/2406 (9.9)	1.85 (1.57-2.17)	1.58 (1.33-1.86)
1 y	1660/8297 (20)	1 [Reference]	382/1544 (25)	1.31 (1.16-1.49)	1.14 (1.00-1.29)	554/2169 (26)	1.37 (1.23-1.53)	1.2 (1.07-1.34)
Other								
LoS >7 d	3068/9570 (32)	1 [Reference]	945/2020 (47)	1.86 (1.69-2.05)	1.71 (1.55-1.88)	1201/2833 (42)	1.56 (1.43-1.70)	1.43 (1.31-1.56)
Discharge delayed >2 d	1127/8787 (13)	1 [Reference]	408/1691 (24)	2.16 (1.90-2.45)	1.97 (1.73-2.24)	490/2406 (20)	1.74 (1.54-1.95)	1.59 (1.41-1.79)
Discharge destination other than usual residence	1413/8635 (16)	1 [Reference]	557/1643 (34)	2.62 (2.33-2.95)	2.35 (2.09-2.65)	700/2358 (30)	2.16 (1.94-2.40)	1.94 (1.74-2.16)
Readmission <30 d	772/8427 (9.2)	1 [Reference]	181/1603 (11)	1.26 (1.06-1.49)	1.18 (0.99-1.40)	244/2344 (10)	1.15 (0.99-1.34)	1.08 (0.92-1.26)

Abbreviations: AOR, adjusted odds ratio; LoS, length of stay; OR, odds ratio.

^a^ Adjusted for age, sex, Charlson Comorbidity Index, and illness severity.

## Discussion

Our findings suggest the feasibility of physician-delivered EHR delirium screening at scale with measured delirium prevalence approaching the likely true prevalence.^4^ This contrasts with lower measured delirium prevalence in routinely acquired data from institutions without formal screening programs^1,2^ or using nurse-led screening.^6^ Limitations include the single center setting impacting generalizability of the findings. Although uncertainty in delirium diagnosis was common, outcomes were similarly poor in those with uncertain and certain delirium, justifying targeted interventions in both groups.

## Notes

Supplement 1.
eMethods

eReferences
**eTable.** Origin of Variables Used in Analysis **eFigure 1.** EHR Cognitive Screen Form **eFigure 2.** Study Flow Diagram. EPR=Electronic Patient Record, Equivalent to Electronic Health Records (EHRs)

Supplement 2.
Data Sharing Statement
