# Supplementary material for: Implementation of Delirium Screening at Scale in Older Patients With Emergency Hospital Admission
Source: JAMA Intern Med. 2025 May 27;185(7):884–7. doi: 10.1001/jamainternmed.2025.1128 (PMC12117490; doi:10.1001/jamainternmed.2025.1128)
Supplement: Supplement 1. — eMethods eReferences eTable. Origin of Variables Used in Analysis eFigure 1. EHR Cognitive Screen Form eFigure 2. Study Flow Diagram. EPR=Electronic Patient Record, Equivalent to Electronic Health Records (EHRs) [file jamainternmed-e251128-s001.pdf]

Supplemental Online Content

Boucher EL, Gan JM, Lovett NG, Smith SC, Shepperd S, Pendlebury ST. Prevalence and outcomes of delirium in older patients with emergency hospital admission. *JAMA Intern Med.* Published online May 27, 2025. doi:10.1001/jamainternmed.2025.1128

eMethods

eReferences

eTable. Origin of Variables Used in Analysis

eFigure 1. EHR Cognitive Screen Form

eFigure 2. Study Flow Diagram. EPR=Electronic Patient Record, Equivalent to Electronic Health Records (EHRs)

This supplemental material has been provided by the authors to give readers additional information about their work.

© 2025 Boucher EL et al. *JAMA Internal Medicine*.

eMethods. Study setting

The Oxford University Hospitals NHS Foundation Trust-OUHFT provides all acute secondary care services to the entire population of the Oxfordshire region (~800,000) as part of the UK-wide publicly funded National Health Service. There is no other provider of acute (emergency) healthcare for the region. Around two thirds of patients aged 70 years or older with emergency hospital admission are managed by the acute general (internal) medicine service (similar to the hospitalist service in the US) led by consultant physicians (equivalent to general internists in the US). Note that general practitioners (or family medicine doctors) do not contribute to this service. The Oxfordshire population is similar to the background population of England as a whole (~95% caucasian at age ≥65 years) with an urban and rural mix although slightly older and less deprived but all levels of deprivation are represented. In 2015, the OUHFT went paperless with the roll-out of electronic health records (EHRs), termed in most NHS hospitals as electronic patient records (EPRs). The EHR was thereafter used to record all healthcare encounters including clinical information, observations performed by nursing staff, laboratory investigations and non-laboratory diagnostic tests (eg radiology, cardiologic investigations, lung function tests, endoscopies etc).

Physician roles and responsibilities in the UK system

The OUHFT runs training programs for residents as do all UK acute hospitals. Delirium is a mandatory curriculum component and delirium is also included in the medical curriculum for all UK medical students. Training programmes for doctors in the UK are long compared to most other countries with more time spent in obtaining general experience in a variety of specialties prior to entering training for a particular specialty. Following graduation from medical school, all UK doctors, irrespective of their ultimate career aims, have to complete two years of foundation training, where they work as junior residents and rotate through a variety of medical and surgical specialities in hospitals in the region. At completion of foundation training, those wishing to become hospital physicians undergo three years of internal medical training (senior residency) where they rotate through various hospital medical specialities before starting training as a specialist registrar (senior specialty resident) in the speciality of their choice (almost always combined with general internist

© 2025 Boucher EL et al. *JAMA Internal Medicine*.

training) for a further five to seven and a half years depending on specialty. At the end of specialty training, they take an exit examination necessary to obtaining a post as a consultant (equivalent to Board certified specialist).

The general (internal) medicine service in the OUHFT is delivered by medical (hospitalist) teams led by consultants and staffed by one senior resident (either a year three internal medical trainee or a specialist registrar) and two more junior residents. At patient admission, residents (both junior and senior) obtain and document the medical history and physical examination (called the “clerking” in the UK), request investigations and write the drug chart with junior residents supervised by the more senior residents or the consultant. Individual medical teams receive new admissions usually on 2-3 days per week (during the day, evening or night) with around 10-30 new patients received each time.

Cognitive screen

In 2010, Pendlebury piloted a cognitive screen for older patients with unplanned admission to acute general (internal) medicine to be completed by the admitting resident doctor. The screen was designed to i) identify cognitive impairment *from whatever cause* through the use of a short cognitive test, ii) identify and document delirium based on the CAM and iii) document pre-existing dementia diagnosis as described previously.[1-3] The cognitive screen was initially delivered by resident doctors using a structured paper history and physical examination form (2012-2015). With the arrival of EHRs (Cerner Millenium), it was implemented as an EHR form, a bespoke electronic form, “built” by the OUHFT EHR team and designed to capture structured clinical data (eFigure-1). Completion of all parts of the electronic form was required ie partial completion of the form was not possible. Our previous work indicated that introduction of the electronic cognitive screen as part of a multicomponent intervention to improve delirium recognition and diagnosis resulted in a six-fold sustained improvement in delirium diagnostic ICD-10 coding (2010-2018).[4]

The short cognitive test used was initially the mini-mental-state-examination (MMSE) but this was subsequently replaced by the shorter and more pragmatic 10-item

© 2025 Boucher EL et al. *JAMA Internal Medicine*.

Abbreviated Mental Test (AMT, range 0-10 points, impaired=≤8). The AMT is highly specific although less sensitive for cognitive impairment defined by the Montreal Cognitive Assessment (MoCA):[1,3] 90% of those with low AMT (≤8) have MoCA<18 (moderate/severe cognitive impairment) compared to only one quarter of those with normal AMT (>8). It should be noted that cognitive tests only identify and quantify objective cognitive deficits - they are not diagnostic tests and cannot discriminate dementia from delirium when used in isolation. They are key however to identifying cognitively impaired patients in whom further assessment and diagnosis as to the cause is required. Cognitive tests are also useful in indicating the severity of impairment (which varies substantially in those with delirium and dementia) and therefore in informing communication with patients and families.

The CAM items were displayed in the electronic form to inform the assessing resident but were not recorded:
A. Acute onset/fluctuation for which collateral history is required.
B. Inattention: Does the patient have difficulty focusing attention (eg did the patient struggle with 20-1 backwards doing the AMT?)
C. Disordered thinking
D. Altered level of consciousness.

Resident doctors based their delirium diagnosis on the CAM items and where these were not all fulfilled completely, or the resident remained otherwise uncertain as to whether delirium was present or not, the diagnosis was recorded as uncertain (indicating possible delirium). Uncertainty in delirium diagnosis may occur even for clinicians with substantial delirium experience and is not unexpected in a fluctuating condition that exists on a spectrum (cf dementia). In addition, the diagnosis is particularly challenging in unfamiliar patients at first assessment particularly where history from family members or caregivers is unavailable since this is key to deciding whether acute onset and fluctuation are present. During the study period, the guidance to clinical staff in our institution was to reassess patients with delirium after 24-48 hours to confirm or refute the diagnosis. However, any reassessment would have been recorded using freetext entries rather than the cognitive screening form and we were therefore unable to assess whether this occurred.

© 2025 Boucher EL et al. *JAMA Internal Medicine*.

The structured format of the electronic cognitive screening form allows the information contained within to be displayed automatically in different locations in the EHR to inform care throughout the pathway. For example, the results (delirium diagnosis, AMT score) populate the EHR observations tab and the “cognitive assessments” tab where all the cognitive screening results over time and across different encounters are shown illustrating the cognitive trajectory. The individual items entered into the electronic screening form are automatically stored along with all other clinical EHR data in the OUHFT data warehouse from where they can be extracted when required for analysis (eTable).[5] For the current study and similar studies, selected data are extracted from the data warehouse by the Information Analysts and uploaded to the Oxford Cognitive Comorbidity, Frailty and Ageing Research Database-Electronic Patient Records (ORCHARD-EPR) by the research team. ORCHARD-EPR is approved by the South Central Oxford C Research Ethics Committee (ref: 23/SC/0258). Patients are not consented for inclusion but there is the possibility for patients to opt-out of being included in the database which is publicised via the OUHFT website and posters around the hospital sites.

Supporting compliance with cognitive screening

Compliance with cognitive screening was maintained by the following actions:[4]

i) Frequency of cognitive screening form completion was highlighted at monthly general (internal) medicine governance meetings;

ii) Reporting of monthly compliance was made to the OUHFT Executive Board including the Chief Executive Officer and the Medical Director;

iii) For the period 2017-2019, compliance was reported nationally as part of the NHS England Dementia Assessment and Referral reporting requirements.

iv) Outstanding cognitive screens were flagged on opening a given patient record in the EHR;

v) Outstanding tasks for completion by medical teams including the cognitive screen were displayed at ward multidisciplinary board rounds;

vi) Individual physicians could view outstanding cognitive screen tasks when looking at the list of patients under their care (via “Worklist”) in the EHR;

© 2025 Boucher EL et al. *JAMA Internal Medicine*.

vii) Ward clerks printed out the list of outstanding tasks including cognitive screens daily which was given to the relevant consultant.

National Early Warning Score

The National Early Warning Score (NEWS) was developed in the UK to improve early detection of clinical deterioration and need for escalation of care (e.g. transfer to the intensive care unit) in hospitalized patients. The NEWS 1 score used in this study includes respiratory rate, oxygen saturation, temperature, systolic blood pressure, heart rate and AVPU (alert, voice, pain, unresponsive) score.[6]

eReferences

1. Pendlebury ST, Klaus SP, Mather M, de Brito M, Wharton RM. Routine cognitive screening in older patients admitted to acute medicine: abbreviated mental test score (AMTS) and subjective memory complaint versus Montreal Cognitive Assessment and IQCODE. *Age Ageing*. 2015;44:1000-5.

2. Pendlebury S, Lovett N, Smith S, et al. Observational, longitudinal study of delirium in consecutive unselected acute medical admissions: age-specific rates and associated factors, mortality and re-admission. *BMJ Open*. 2015;5:e007808-e007808.

3. Emery A, Wells J, Klaus SP, Mather M, Pessoa A, Pendlebury ST. Underestimation of Cognitive Impairment in Older Inpatients by the Abbreviated Mental Test Score versus the Montreal Cognitive Assessment: Cross-Sectional Observational Study. *Dement Geriatr Cogn Dis Extra*. Sep-Dec 2020;10:205-215.

4. Pendlebury ST, Lovett NG, Thomson RJ, Smith SC. Impact of a system-wide multicomponent intervention on administrative diagnostic coding for delirium and other cognitive frailty syndromes: observational prospective study. *Clinical Medicine*. 2020;20:454-464.

© 2025 Boucher EL et al. *JAMA Internal Medicine*.

5. Boucher E, Jell A, Singh S, et al. Protocol for the Development and Analysis of the Oxford and Reading Cognitive Comorbidity, Frailty and Ageing Research Database-Electronic Patient Records (ORCHARD-EPR). *BMJ Open*. 2024;14:e085126.

6. Smith GB, Prytherch DR, Meredith P, Schmidt PE, Featherstone PI. The ability of the National Early Warning Score (NEWS) to discriminate patients at risk of early cardiac arrest, unanticipated intensive care unit admission, and death. *Resuscitation*. 2018;84:465-70.

© 2025 Boucher EL et al. *JAMA Internal Medicine*.

eTable. Origin of variables used in analyses.

	Resident doctors	Nursing staff	Laboratory staff	Administrative coding staff	Hospital Information Analysts	ORCHARD-EPR Research Team
Cognitive screen (AMT and delirium diagnosis)*	✓					
Observations*		✓				
Falls*		✓				
Incontinence*		✓				
Pressure sore risk (Braden)*		✓				
Laboratory tests			✓			
Primary and secondary diagnoses (ICD-10 codes)*				✓		
CCI (derived from ICD-10 codes)						✓
Frailty (HFRS derived from ICD-10 codes)						✓
NEWS (derived from observations)						✓
Infection diagnosis (derived from ICD-10 codes)						✓
Length of stay (calculated from admission and discharge dates					✓	
Discharge destination*					✓	
Readmission						✓
Mortality (date of death)					✓	
Time to death						✓

*Entered into EHR as part of standard clinical care. AMT=Abbreviated Mental Test, CCI=Charlson Comorbidity Index, HFRS=Hospital Frailty Risk Score, NEWS=National Early Warning Score

© 2025 Boucher EL et al. *JAMA Internal Medicine*.

eFigure-1. EHR Cognitive Screen form

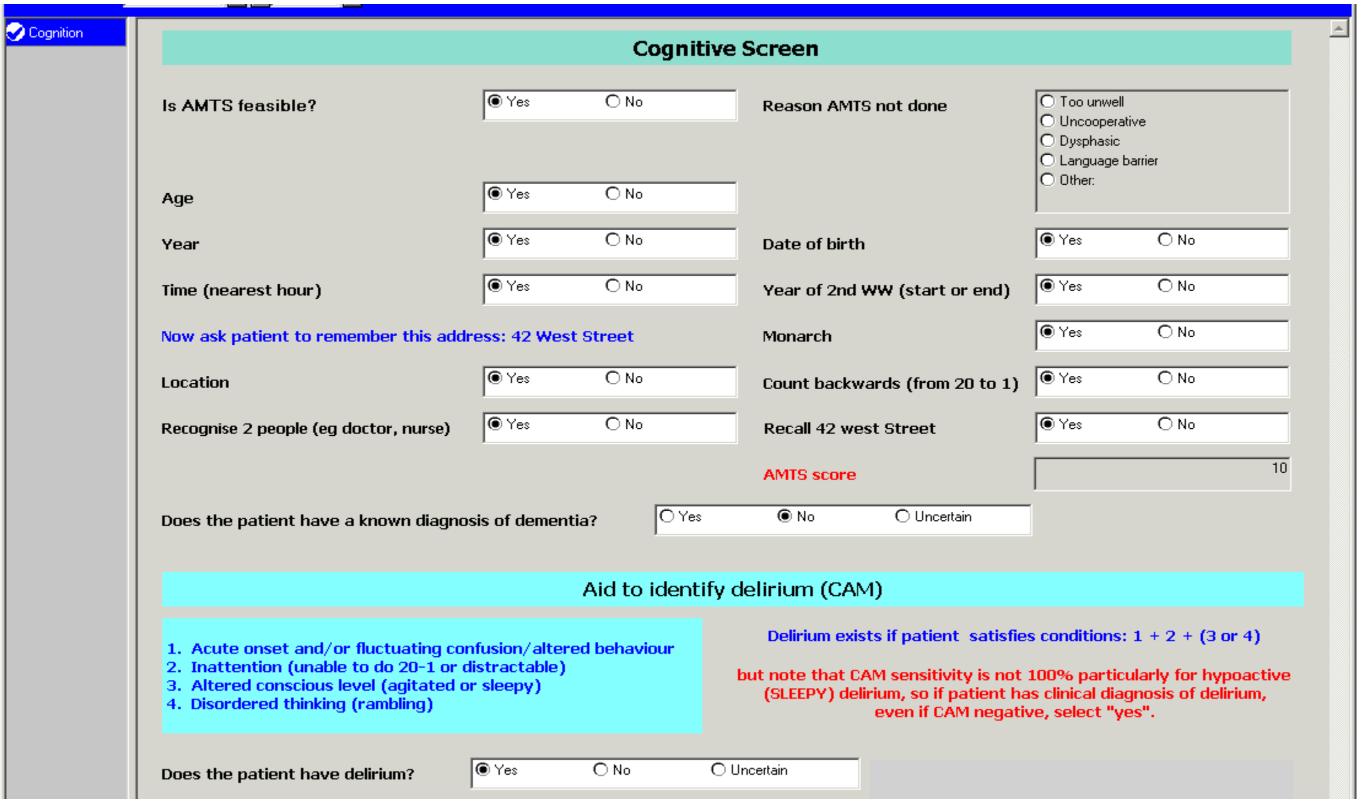
Cognition

Cognitive Screen

Is AMTS feasible? (•) Yes ( ) No

Reason AMTS not done ( ) Too unwell ( ) Uncooperative ( ) Dysphasic ( ) Language barrier ( ) Other:

Age (•) Yes ( ) No

Year (•) Yes ( ) No

Date of birth (•) Yes ( ) No

Time (nearest hour) (•) Yes ( ) No

Year of 2nd WW (start or end) (•) Yes ( ) No

Now ask patient to remember this address: 42 West Street

Monarch (•) Yes ( ) No

Location (•) Yes ( ) No

Count backwards (from 20 to 1) (•) Yes ( ) No

Recognise 2 people (eg doctor, nurse) (•) Yes ( ) No

Recall 42 west Street (•) Yes ( ) No

AMTS score 10

Does the patient have a known diagnosis of dementia? ( ) Yes (•) No ( ) Uncertain

Aid to identify delirium (CAM)

1. Acute onset and/or fluctuating confusion/altered behaviour
2. Inattention (unable to do 20-1 or distractable)
3. Altered conscious level (agitated or sleepy)
4. Disordered thinking (rambling)

Delirium exists if patient satisfies conditions: 1 + 2 + (3 or 4)

but note that CAM sensitivity is not 100% particularly for hypoactive (SLEEPY) delirium, so if patient has clinical diagnosis of delirium, even if CAM negative, select "yes".

Does the patient have delirium? (•) Yes ( ) No ( ) Uncertain

© 2025 Boucher EL et al. *JAMA Internal Medicine*.

eFigure-2. Study Flow Diagram. EPR=Electronic Patient Record, equivalent to Electronic Health Records (EHRs).

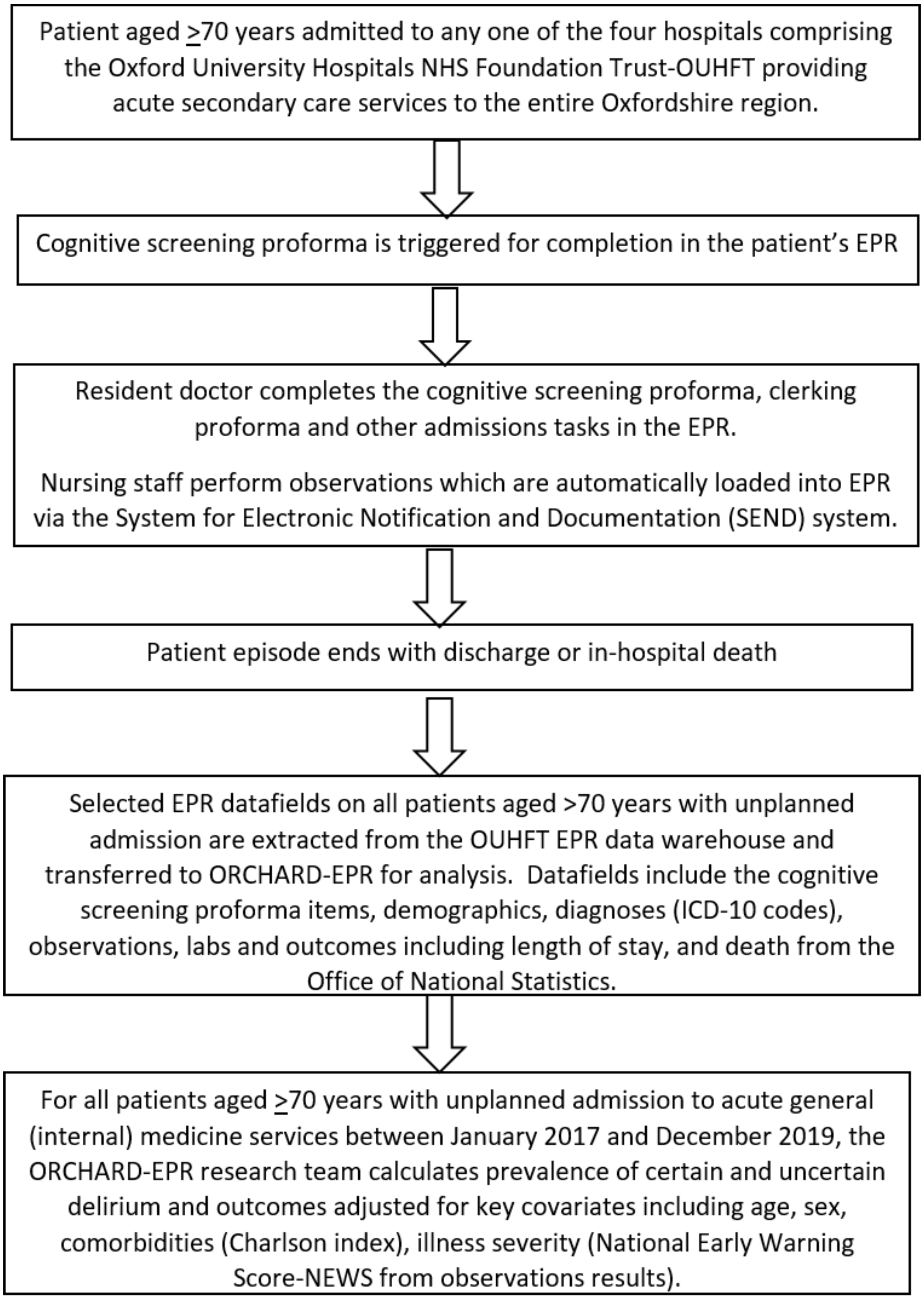

© 2025 Boucher EL et al. *JAMA Internal Medicine*.

© 2025 Boucher EL et al. *JAMA Internal Medicine*.
