# Supplementary material for: Implementation of Delirium Screening at Scale in Older Patients With Emergency Hospital Admission
Source: JAMA Intern Med. 2025 May 27;185(7):884–7. doi: 10.1001/jamainternmed.2025.1128 (PMC12117490; doi:10.1001/jamainternmed.2025.1128)
Supplement: Supplement 2. — Data Sharing Statement [file jamainternmed-e251128-s002.pdf]

Data Sharing Statement

Boucher. Implementation of Delirium Screening at Scale in Older Patients With Emergency Hospital Admission. *JAMA Intern Med*. Published May 27, 2025.
doi:10.1001/jamainternmed.2025.1128

Data
Data available: No

Additional Information
Explanation for why data not available: At present, ethics approval restrictions limit the use of data to researchers within the University of Oxford or Oxford University Hospitals NHS Foundation Trust. Requests for data should be made to Professor Sarah Pendlebury (sarah.pendlebury@ndcn.ox.ac.uk) and will be considered on a case by case basis.
